# Genotyping-by-sequencing provides new genetic and taxonomic insights in the critical group of *Centaurea tenorei*

**DOI:** 10.3389/fpls.2023.1130889

**Published:** 2023-05-16

**Authors:** Daniele De Luca, Emanuele Del Guacchio, Paola Cennamo, Luca Paino, Paolo Caputo

**Affiliations:** ^1^ Department of Biology, University of Naples Federico II, Naples, Italy; ^2^ Botanical Garden of Naples, University of Naples Federico II, Naples, Italy; ^3^ Department of Humanities, University Suor Orsola Benincasa, Naples, Italy

**Keywords:** Asteraceae, hybridization, mixed ploidy, population structure, SNPs

## Abstract

*Centaurea* L. is one of the most widespread, differentiated, and critical genera of Asteraceae in the Euro-Mediterranean area, with more than 100 currently recognized species inhabiting the region. The controversial *C. tenorei* group, narrowly endemic to the Peninsula of Sorrento (Campania region, southern Italy), includes three weakly differentiated microspecies: *C. tenorei* Guss. ex Lacaita, *C. montaltensis* (Fiori) Peruzzi and *C. lacaitae* Peruzzi. However, their taxonomic distinctiveness and relationships with close or sympatric species are still unclear. In particular, the existence in several localities of individuals with intermediate morphology suggests inadequate taxonomic assessment within the group or hybridization and introgression with other species. In this study we aimed at defining population structure in this complex. With this objective, we sampled the three currently accepted species from their *loci classici* (i.e., the localities in which the taxa were originally described) and from other localities throughout the range, including populations of difficult identification occurring where the ranges of different taxa overlap. We employed a panel of SNPs obtained via genotyping-by-sequencing for investigations on genetic structure, admixture and ploidy inference, the latter also compared with chromosome counts. Our results showed that *Centaurea tenorei* s.l. is consistently tetraploid, contradicting the current taxonomy that was also based on ploidy level. Population structure analyses indicated the presence of four to seven clusters, most of which with clear evidence of admixture. Furthermore, contrarily to what previously supposed, we demonstrated a remarkable contribution of *C. deusta*, more that of *C. cineraria* in the genetic make-up of *C. tenorei*. However, we found a population of *C. cineraria* outside its ecological range, probably driven by climate change, which could be responsible in the future of further hybridization phenomena.

## Introduction

Taxonomy and phylogeny of genus *Centaurea* L. have been intensely investigated in the last years, from both a morphological and molecular point of view (e.g., Font et al., 2002; Garcia-Jacas et al., 2006; Suarez-Santiago et al., 2007; Garcia-Jacas et al., 2009; Hilpold et al., 2011; Hilpold et al., 2014b; López-Alvarado et al., 2014; Ranjbar and Negaresh, 2014; Bona, 2015; Arnelas et al., 2018; Arnelas et al., 2020; Del Guacchio et al., 2020a; Novaković et al., 2022). Various of these contributions show that patterns of descent in this genus do not mirror the current taxonomy and indeed are more often associated to geographical proximity; this has been interpreted as the result of diffuse hybridization/introgression and gene flow between divergent populations, species and even sections in a context of overall low molecular variation or of Incomplete Lineage Sorting (ILS), complicated by convergence in diagnostic morphological traits (Suarez-Santiago et al., 2007; Hilpold et al., 2014a). As a consequence, species sampled in multiple populations are “almost never monophyletic” (Hilpold et al., 2014b, p. 205), incongruence in gene trees is rampant, and phylogeny does not necessarily correspond to systematics (Hilpold et al., 2014b; López-Alvarado et al., 2014; Novaković et al., 2022).

Hybridization indeed appears to be a significant evolution driver in the genus (Garcia-Jacas et al., 2009; Hilpold et al., 2014b; López-Alvarado et al., 2014; Arnelas et al., 2018), especially at the homoploid level (Font et al., 2009; Pisanu et al., 2011; Mameli et al., 2014). In addition, polyploids can be found, as different cytotypes within the same, often widely distributed species (López-Alvarado et al., 2014). Moreover, they may be involved in the origin of new species (Garcia-Jacas, 1998; Koutecký, 2007; Garcia-Jacas et al., 2009; Mráz et al., 2012; Merle et al., 2022), or even in the appearance of highly invasive hybrids (Blair and Hufbauer, 2010). All these phenomena (i.e., hybridization, ILS, low molecular variation, convergence, intraspecific ploidy variation, polyploid origin of species) generate pervasive incongruence between phylogenetic hypotheses, and poorly supported trees (Hilpold et al., 2014a; López-Alvarado et al., 2014); this in turn greatly complicates taxa delimitation (e.g., Novaković et al., 2022) and phylogenetic inference (e.g., Hilpold et al., 2014b).

One of the taxonomically critical groups with mixed ploidy within *Centaurea* is that of *C. tenorei* Guss. ex Lacaita. According to the latest reassessment (Peruzzi and Scoppola, 2008), the group (basic chromosome number x = 9) is represented by three species, namely *C. lacaitae* Peruzzi (4x; 2n = 36)*, C. montaltensis* (Fiori) Peruzzi (4x; 2n = 36), and *C. tenorei* s.str. (2x; 2n = 18), all narrow endemic to the Sorrento Peninsula (Campania, south-western Italy). As indicated in Santangelo et al. (2017), these taxa are remarkably variable and weakly differentiated in terms of morphology, ecology and distribution (Lacaita, 1922; Pignatti and Lausi, 1982; Guarino and Rampone, 2006). As a consequence, taxonomy of the group has greatly varied along time (Santangelo et al., 2017 and references therein), and it is still unsatisfactory (PFI [Portale della Flora d’Italia], 2022), because assignment of some individuals to one of the three species remains difficult (Santangelo et al., 2017). Interpretation of relationships and boundaries in this group is additionally complicated by the sympatric occurrence of two further taxa in the Peninsula of Sorrento, i.e., *C. deusta* Ten. subsp. *deusta* and *C. cineraria* L. subsp. *cineraria* (both mostly diploids with 2n = 18: Bedini and Peruzzi, 2021), which are said or presumed to form natural hybrids with *C. tenorei* s.l. (Del Guacchio et al., 2020a). The systematic reassessment of this group is not only challenging, but also relevant by a conservational point of view. In fact, it is protected throughout its range at regional level because regarded as endangered but data for taxonomic assessment (and consequent conservation) are deficient and also afflicted by its obscure taxonomy (Rossi et al., 2020).

The study of population structure is a key prerequisite for detection of lineages and/or evolutionary significant units and their phylogenetic potential (Allendorf et al., 2013), as well as for taxonomic recognition of weakly defined taxa or identification of dubious specimens (De Castro et al., 2020; Favre et al., 2021). Moreover, knowledge on population structure is essential for setting conservation priorities and strategies (Wuyun et al., 2015; Kienberg and Becker, 2017; Hatmaker et al., 2018; Cheng et al., 2020). The genus *Centaurea*, however, may be difficult to describe in terms of significant evolutionary units, population structure or phylogeny, especially with traditional molecular techniques (Herrando-Moraira et al., 2019). Higher resolution methods, as those involving the use of a great number of Single Nucleotide Polymorphisms (SNPs) via Next Generation Sequencing (NGS) may be regarded as the elective choice for such problems (e.g., Paetzold et al., 2019; Hyun et al., 2020). However, the different ploidy levels occurring in our chosen system may represent a difficulty. In plants, in facts, inference of population structure is often complicated by the occurrence of mixed-ploidy populations (Kolář, 2021), which occur in about 16% of plant species (Rice et al., 2015). In spite of the significance of ploidy variation in plants, however, the number of software tools available for routine population genomic analyses in polyploids and, especially, in mixed-ploidy systems, is comparatively low in respect to diploids (Dufresne et al., 2014; Kolář, 2021); one of the few examples of such tools for NGS SNPs data is fineRADStructure (Malinsky et al., 2018). On the other hand, mixed-ploidy systems allow the study of possible correlation between ploidy and genetic variability within populations or among single individuals (Soltis et al., 2016). Recently new techniques and accompanying software, are capable of inferring this correlation directly from NGS data (Gompert and Mock, 2017; Weiß et al., 2018; Soraggi et al., 2022).

In this study, using a NGS approach, we aim at (a) describing genetic structure in populations of *C. tenorei* group, (b) testing the occurrence of admixture within the *C. tenorei* species group or between these latter and *C. cineraria* or *C. deusta*, (c) detect a possible correlation between ploidy and populations \ taxa, (d) understanding whether genetic variation among our three taxa of interest is indeed sufficiently discontinuous to grant their taxonomic recognition.

## Materials and methods

### Selection of taxa, sample collection and DNA extraction

**Figure 1:**
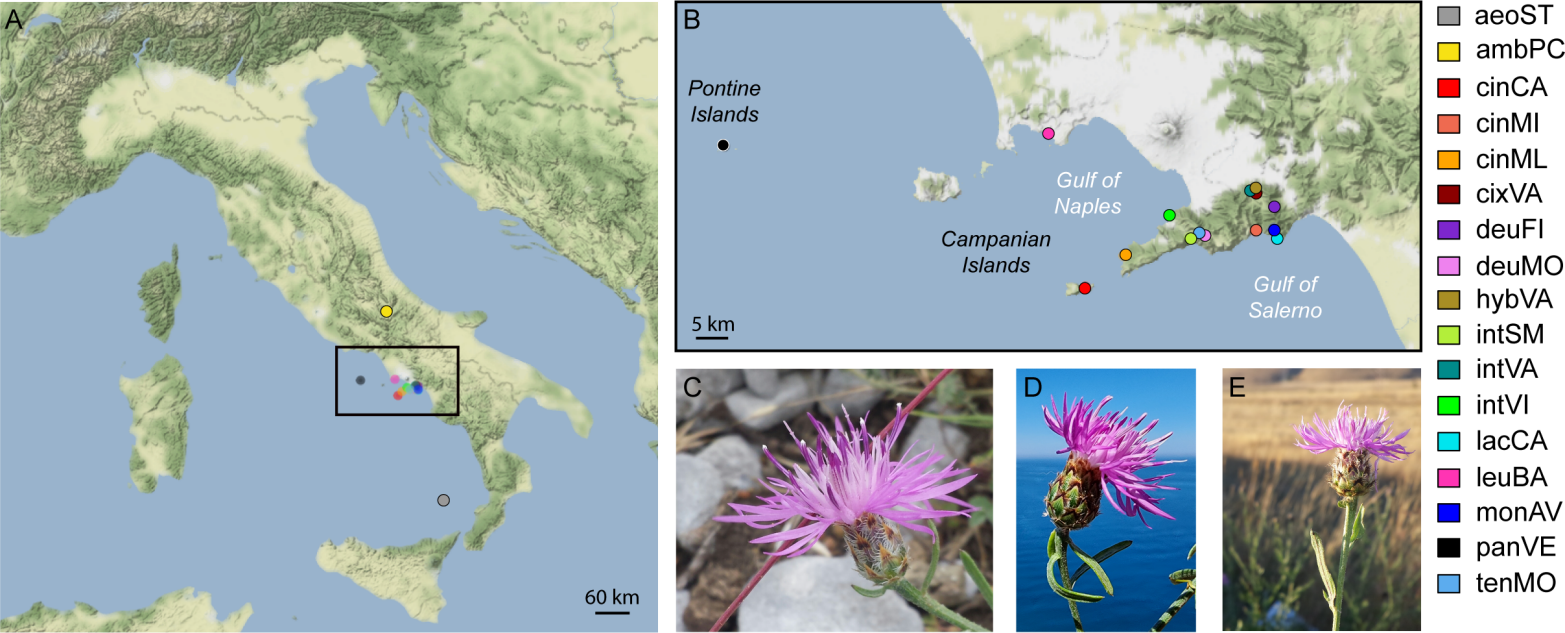
Distribution of sampling localities. **(A)** sampling points; **(B)** zoom of sampling points in Campania region (Italy); **(C)** flowering head of *C. montaltensis* (Mt. Avvocata, Salerno Province); **(D)** flowering head of *C. lacaitae* (Vico Equense, Naples Metropolitan City); **(E)** flowering head of *C. ambigua* subsp. *ambigua* (Piano delle Cinque Miglia, Rocca Pia, Aquila province). Abbreviations in legend are as in **Table 1**.

We included in this study the populations of the three microspecies of the group (see codes in **Table 1** and **Figure 1**), i.e., *C. lacaitae* [lacCA], *C. montaltensis* [monAV], and *C. tenorei* [tenMO] from the localities in which the taxa were originally described and are currently found. Some populations, difficult to ascribe to a single taxon, were included as well: some atypical individuals of *C. cineraria* from Valico di Chiunzi with glabrescent leaves [cixVA]; a single presumed hybrid between the former taxon and *C. deusta* [hybVA]; several populations showing, at various degrees, intermediate features between *C. tenorei* s.l. and *C. cineraria* (cf. Lacaita, 1922; Santangelo et al., 2017; Del Guacchio et al., 2020a): Santa Maria del Castello [intSM], Vico Equense [intVI], and again Valico di Chiunzi [intVA]. We also included some populations of two taxa known or much suspected to hybridize with *C. tenorei* s.l. (Del Guacchio et al., 2020a): two populations of *C. deusta* subsp. *deusta* from Mt. Molare [deuMO] and Mt. Finestra [deuFI], and three populations of *C. cineraria* subsp. *cineraria*, gathered from Minori [cinMI], Marina della Lobra [cinML], and Capri Island [cinCA].

**Table 1:** List of taxa sampled in the present study, with indications of localities, geographic coordinates, voucher specimen preserved at NAP and number of individuals collected.

Taxon	Locality (province, region)	Coordinates	Population voucher specimen	Individuals	Code
*C. lacaitae* Peruzzi	Capo d’Orso (Salerno, Campania) *	40.636 N 14.678 E	NAP0001741	9	lacCA
*C. montaltensis* (Fiori) Peruzzi	Mt. Avvocata (Salerno, Campania) *	40.653 N 14.669 E	NAP0001750	16	monAV
*C. tenorei* Guss. ex Lacaita	Mt. Molare (Naples, Campania) *	40.648 N 14.501 E	NAP0001746	7	tenMO
Possible intermediates between *C. cineraria* and *C. tenorei*	Vico Equense (Naples, Campania)	40.677 N 14.436 E	NAP0001744	7	intVI
	Santa Maria del Castello (Naples, Italy)	40.639 N 14.479 E	NAP0001737	6	intSM
	Valico di Chiunzi, (Salerno. Italy)	40.718 N 14.618 E	NAP0001743	5	intVA
*C. cineraria* L. subsp. *cineraria*	Capri (Naples, Campania)	40.553 N 14.243 E	NAP0001745	5	cinCA
	Marina della Lobra (Naples, Campania)	40.608 N 14.333 E	NAP0001748	5	cinML
	Minori (Salerno, Campania)	40.650 N 14.627 E	NAP0001738	7	cinMI
*Centaurea* cf. *cineraria*	Valico di Chiunzi (Salerno, Campania)	40.717 N 14.631 E	NAP0001751	7	cixVA
*C. deusta* Ten. subsp. *deusta*	Mt. Finestra (Salerno, Campania)	40.692 N 14.669 E	NAP0001739	5	deuFI
	Mt. Molare (Naples, Campania)	40.648 N 14.501 E	NAP0001740	5	deuMO
*C. deusta* subsp*. leucolepis* (DC.) Del Guacchio, Cennamo & P.Caputo	Bagnoli (Naples, Campania) *	40.815 N 14.162 E	NAP0001742	3	leuBA
Possible *C. deusta* × *C.* cf. *cineraria*	Valico di Chiunzi (Salerno, Campania)	40.719 N 14.616 E	—	1	hybVA
*C. aeolica* Guss. ex Lojac.	Stromboli Island (Messina, Sicily) *	38.796 N 15.237 E	NAP0001791	1	aeoST
*C. ambigua* Guss. subsp. *ambigua*	Piano delle Cinque Miglia (L’Aquila, Abruzzo)	41.888 N 14.000 E	NAP0001749	2	ambPC
*C. pandataria* (Fiori & Bég.) Bég.	Ventotene Island (Latina, Latium) *	40.796 N 13.428 E	NAP0001747	5	panVE

An asterisk indicates localities cited in the protologues of the species.

For comparison purposes, we employed one population of the other subspecies of *C. deusta*, i.e., *C. deusta* subsp. *leucolepis* (DC.) Del Guacchio, Cennamo & P.Caputo (= *C. corensis* Vals. & Filigh.) [leuBA], occurring in the same region but not sympatric with our group of interest. We also added two individuals of *C. ambigua* Guss. subsp. *ambigua* [ambPC] because, even if *C. ambigua* s.l. is completely allopatric, it is often included in the taxonomic aggregate of *C. tenorei* (Santangelo et al., 2017), and indeed regarded by Guadagno (1932) as an ancestor of it. As outgroups, we employed a population of the allopatric taxon *C. pandataria* (Fiori & Bég.) Bég. [panVE] (for the correct authorship of the name and its relationship with *C. cineraria*, see Del Guacchio et al., 2022 but cf. also Turland et al., 2018, art. 46.2), and an individual of *C. aeolica* Guss. ex Lojac. [aeoSTR], very close to the former species (Del Guacchio et al., 2022 and references therein).

We field-collected leaf material from 96 samples of the above-mentioned taxa (**Table 1**, **Figure 1**). The only exception was *C. aeolica*, for which we employed material from a plant cultivated at the Botanic Garden of Naples and originated from seeds collected in the wild. We collected 4–7 individuals per population (see **Table 1**), with the exception of hybVA (only a single individual found) and some outgroups (*C. aeolica* and *C. ambigua*). We gathered a larger number of samples for monAV because this population extends broadly along an altitudinal cline (600–1000 m a.s.l.), and we aimed at checking whether any genetic difference might occur within it. The taxa were identified mainly by using Peruzzi and Scoppola (2008) and Pignatti (2018). Voucher specimens are deposited at the Herbarium of the Botanical Garden of Naples (NAP). Samples were taken during flowering time (late spring-summer) and leaf material was stored at −80°C until DNA extraction. DNA extraction was carried out using the GeneAll® Exgene™ Plant SV mini kit (GeneAll Biotechnology, Seoul, Korea) following manufacturer’s protocol. To obtain high-quality DNA, frozen leaves were grinded in liquid nitrogen. Integrity of extracted DNA was evaluated on 0.8% gel electrophoresis and concentration was determined with Qubit® dsDNA HS Fluorometer assay (Invitrogen, ThermoFisher Scientific, Waltham, MA, USA).

### Genomic library preparation and SNP discovery and genotyping

We performed genotyping-by-sequencing (GBS) for *de novo* SNP detection. Libraries were prepared using MsII restriction enzyme (mean insert size of about 200 bp; recognition sequence and cut site, ↑↓: CAYNN↑↓NNRTG) and sequenced at LGC Biosearch Technologies (Berlin, Germany) on a 2 × 150 bp Illumina NextSeq® 550 platform (Illumina, San Diego, CA, USA). Library groups were demultiplexed into samples using the Illumina bcl2fastq v2.17.1.14 software (available at https://support.illumina.com/downloads/bcl2fastq-conversion-software-v2-20.html) according to their inline barcodes and verification of restriction site (no mismatches or Ns were allowed in the inline barcodes, but Ns were allowed in the restriction site). Sequencing adapters were clipped from all reads, reads with final length < 20 bp were discarded and reads with 5’ ends not matching the restriction site were removed as well. Reads were further filtered by quality, trimming at 3’ end to get a Phred quality score ≥ 20 over a window of ten bases and discarding reads with a final length < 20 bp. Forward and reverse reads were assembled in contigs using BBMerge v34.48 (Bushnell et al., 2017). Unique (non-redundant) combined reads were obtained with CD-HIT-EST v4.6.1 (Li and Godzik, 2006) allowing up to 5% differences and excluding singletons and clusters containing < 20 reads. In absence of a reference genome for any of the taxa of interest, alignment of quality trimmed reads was carried out using a cluster reference in BWA-MEM v0.7.12 (Li and Durbin, 2009). SNP discovery and genotyping were performed with Freebayes v1.0.2-16 (Garrison and Marth, 2012) at the following parameters: min-base quality = 10, min-supporting-allele-qsum = 10, read-mismatch-limit = 3, min-coverage = 5, no-indels, min-alternate-count = 4, exclude-unobserved-genotypes, genotype-qualities, no-mnps, no-complex, mismatch-base-quality-threshold = 10). This analysis was initially run twice, separately setting ploidy to 2 and 4 according to chromosome numbers known from literature for some of the investigated species. SNPs were then filtered under the following rules: variants had to exceed 8 reads per locus, genotypes had to be observed in at least 66% of samples, and the minimum allele frequency across samples should exceeded 5%. After results of ploidy estimation using NGS data (see below), we also generated a dataset with triploid counts.

### Ploidy estimation from NGS data and chromosome counts

To estimate the ploidy of single individuals, we analyzed the GBS data using the software nQuire (Weiß et al., 2018). This software models the distribution of base frequencies of SNPs from BAM files using a Gaussian Mixture Model, compares it with the expected distribution in diploids, triploids and tetraploids, and uses a maximum likelihood approach to select the most plausible ploidy model. We used a minimum frequency of 0.2 for segregating bases (-f option), a minimum coverage of 10 (-c option) and a minimum mapping quality of 30 (-q option). Despite the stringent filters used, we also ran the “denoising” subcommand to reduce background noise in base frequency histograms due to mismappings, as suggested by the software manual. This subcommand uses a Gaussian Mixture Model with Uniform noise component (GMMU) (Weiß et al., 2018) to assess the extent of this uniform noise and scales it down to easily detect peaks in the histogram of base frequencies. The fixed model with the smallest Δlog*L* is chosen as the true ploidy level. After the results of this analysis, we detected some triploids and, therefore, recalled SNPs in FreeBayes setting ploidy also to 3.

We compared such estimated ploidy values with chromosome counts for the following populations: aeoST, cinML, intSM, intVA, leuBA, lacCA, monAV, panVE, and tenMO. For this purpose, we employed cypselae deposited in the seed bank of the Botanic Garden of Naples or collected in field. Chromosomal observations were made on root tips obtained from the germination of cypselae in 1% agar Petri dishes. After the appearance of root tips, plates were stored for 12-24 hours at 4°C. Then, seeds with attached root tip were immersed in 0.4% colchicine for 4 hours and subsequently fixed in Carnoy fixative solution for 1 hour. After hydrolysis in 1N HCl at 60° C for 7 min, the tips were stained with Schiff’s fuchsin-sulfite reagent (Sigma-Aldrich by Merck, St. Louis, MO, USA). Root tips were then soaked in 45% acetic acid, macerated and squashed. Metaphasic plates were observed, whenever possible, from root tips of 3-5 different individuals from seeds of at least 3 different mother-plants, using a Nikon Eclipse Ci-L microscope (Nikon, Tokyo, Japan).

### Data filtering and population structure analyses

The final dataset containing mixed-ploidy individuals was further subject to filtering by missingness and linkage to reduce their impact of subsequent analyses. Specifically, we first calculated the missingness per locus, then ordered the loci by increasing missingness and retained, when multiple SNPs occurred in the same contig, only the one with less missing information across most of individuals. After this first selection, we filtered the dataset by missingness per individual, removing all the samples with missing data < 50%, and, per locus, removing all loci with ≥ 30% of missing data. To assess if the selected loci were neutral, in absence of a pipeline/software capable of handling data with mixed ploidy, we manually created an input file for BayeScan v2.1 (Foll and Gaggiotti, 2008) making all samples of the same ploidy (tetraploid) by adding one or two columns of missing data to triploids and diploids, respectively. The analysis was run with prior odds set to 100 and the other values as default. Convergence of runs and loci under selection were assessed following the tutorial available at http://evomics.org/wp-content/uploads/2016/01/BayeScan_BayeScEnv_exercises.pdf.

As first exploratory analysis of structure in our data, we carried out a principal component analysis (PCA) in adegenet v2.1.5 (Jombart, 2008; Jombart and Ahmed, 2011). After creating a ‘genlight’ object following the instructions in the package manual, we used the function ‘glPca’ to implement the analysis accommodating different levels of ploidy across samples in the data. We also used the function ‘find.clusters’ to group our samples in clusters based on their genetic similarity. This function first transforms the data using PCA (after specifying the number of retained PCs) and then choses the optimal number of clusters using the Bayesian information criterion (BIC). Estimates of allele frequency distribution and of expected (He) and observed heterozygosity (Ho) per locus were conducted in the same package using the functions ‘gl.Mean’, ‘gl.He’, and ‘gl.Ho’ respectively. Putative significant differences between He and Ho were evaluated with the Bartlett’s test of homogeneity of variances.

To assess population structure and detect recent shared ancestry among population samples, we used fineRADstructure (Malinsky et al., 2018), a model-based Bayesian clustering approach that works with mixed-ploidy datasets from unmapped SNPs. In this approach, co-ancestry is determined calculating the number of SNPs shared by individuals (the “co-ancestry proportion”), and then assigning individuals with similar co-ancestry values to the same genetic cluster. We ran the analysis following the instructions provided on the software webpage (https://www.milan-malinsky.org/fineradstructure), also ordering loci according to linkage disequilibrium (script ‘sampleLD.R’) as suggested by the author for unmapped loci.

To evaluate the genetic make-up of triploid individuals discarded from previous analyses for their high degree of missingness, we selected a panel of 267 SNPs with locus missingness ≤ 0.03% and, retaining all the original 96 individuals (diploids, triploids, and tetraploids), we carried out an analysis in STRUCTURE v2.3.4 (Pritchard et al., 2000). This software was chosen over others commonly used in population assignation and admixture detection (e.g., ADMIXTURE (Alexander et al., 2009), fastSTRUCTURE (Raj et al., 2014)) because of its capability and robustness at handling datasets with mixed-ploidy populations (Stift et al., 2019). To estimate the number of genetic clusters (*K*), STRUCTURE was run with *K* varying from 2 to 17 for 100,000 generations, the first 50,000 of which were discarded as burn-in. Each value of *K* was evaluated using ten independent Markov Chain Monte Carlo (MCMC) replicates. To choose the best value of *K*, we looked at the plot showing the log probability of the data [ln Pr(X|K)] against a range of K values as provided by STRUCTURE HARVESTER (Earl and vonHoldt, 2012) and selected the K after which the trend of ln Pr(X|K) reaches a plateau (Pritchard et al., 2000, Pritchard et al., 2010; Gilbert et al., 2012). We chose this method over that of Evanno (Evanno et al., 2005) because it has been demonstrated that the latter is strongly biased towards K=2 (Janes et al., 2017). The results of the ten MCMC iterations of each K obtained from STRUCTURE HARVESTER were combined and analysed in CLUMPAK (Kopelman et al., 2015) using LargeKGreedy algorithm (Jakobsson and Rosenberg, 2007) and visualised in the same webserver using DISTRUCT (Rosenberg, 2004).

Genetic differentiation among population groups identified by previous analyses was calculated using the Fixation index (F_ST_) (Weir and Cockerham, 1984) in the R package StAMPP (Pembleton et al., 2013). Population samples constituted by 1-2 individuals (i.e., aeoST, ambPC, HybVA) were removed from the analysis. Significance of pairwise F_ST_ values was determined after 1000 replications.

## Results

### Ploidy estimation and chromosome count

We obtained from SNP data analysis (through nQuire software) the following estimation for each taxon (see **Table 2** for the single populations, complete data in **Supplementary Table 1**): *C. aeolica* (2n = 36), *C. ambigua* subsp. *ambigua* (2n = 27, 36), *C. deusta* subsp. *deusta* (2n = 18), *C. deusta* subsp. *leucolepis* (2n = 36), *C. lacaitae* (2n = 27, 36), *C. montaltensis* (2n = 27, 36), *C. pandataria* (2n = 18), *C. tenorei* (2n = 27, 36). Intermediate populations (intSM, intVA, intVI) resulted tetraploid, or rarely triploids (see **Table 2** for further details). Metaphasic chromosome counts were obtained for 9 populations (**Table 2**) and returned the same results of NGS estimation, except for *C. aeolica*, which was counted as diploid (2n = 18).

**Table 2:** Ploidy of taxa under investigation estimated by chromosome counting and NGS data.

Taxon	Population	Count	Ploidy from NGS	Literature
*C. lacaitae*	lacCA	2n = 36	tetraploid (triploid)	2n = 36
*C. montaltensis*	monAV	2n = 36	tetraploid (triploid)	2n = 36
*C. tenorei*	tenMO	2n = 36	tetraploid (triploid)	2n = 18
Possible intermediates between *C. cineraria* and *C. tenorei*	intSM	2n = 27	tetraploid (triploid)	–
	intVA	2n = 36	tetraploid (triploid)	–
	intVI	–	tetraploid (rarely triploid)	–
*C. cineraria* subsp. *cineraria*	cinCA	–	diploid	2n = 18, 36
	cinMI	2n = 18	diploid (rarely tetraploid)	
	cinML	–	diploid	
*Centaurea* cf. *cineraria*	cixVA	–	diploid	–
*C. deusta* subsp. *deusta*	deuFI	–	diploid	2n = 18
	deuMO		diploid	
*C. deusta* subsp*. leucolepis*	leuBA	2n = 36	tetraploid	2n = 36
*C. deusta* hybrid	hybVA	–	diploid	–
*C. aeolica*	aeoST	2n = 18	tetraploid	2n = 18
*C. ambigua* subsp. *ambigua*	ambPC	–	triploid/tetraploid	2n = 36
*C. pandataria*	panVE	2n = 18	diploid	2n = 18

Literature data are retrieved from Bedini and Peruzzi (2021). No viable root tip was obtained for *C. deusta*.

### Overview of GBS data

Illumina sequencing returned ~2.7 million reads per sample, for a total of 205,140 loci (~13× coverage). Of these loci, 130,411 were polymorphic and contained 466,078 SNPs. After filtering procedures described in Materials and Methods, we obtained two datasets: one, including 7,473 loci and 75 individuals and utilized for PCA, fineRADstructure, and F_ST_ inferences (**Supplementary Table 2**); a second one, with 267 loci and all 96 individuals for STRUCTURE analysis (**Supplementary Table 3**). From the first dataset, one individual of *C. ambigua*, two individuals of *C. deusta* (from Mt. Molare) and one intermediate individual (from intVI), as well as all the triploid samples were discarded because afflicted by high individual missingness (mean = 0.68). BayeScan analysis identified only 37 loci out of 7473 under positive selection (qval < 0.05) (**Supplementary Table 4**), which were kept in the final dataset for subsequent analyses, after verifying that the results of an exploratory PCA were completely overlapping to those of the full dataset.

Mean observed heterozygosity was 0.120, significantly lower (p < 0.001) than expected heterozygosity (= 0.285) (**Supplementary Figures 1** and **2**). Most of loci contained alleles with frequency between 0.2 and 0.9, and only a limited portion of them was fixed (**Supplementary Figure 3**).

### Population structure and genetic differentiation

**Figure 2:**
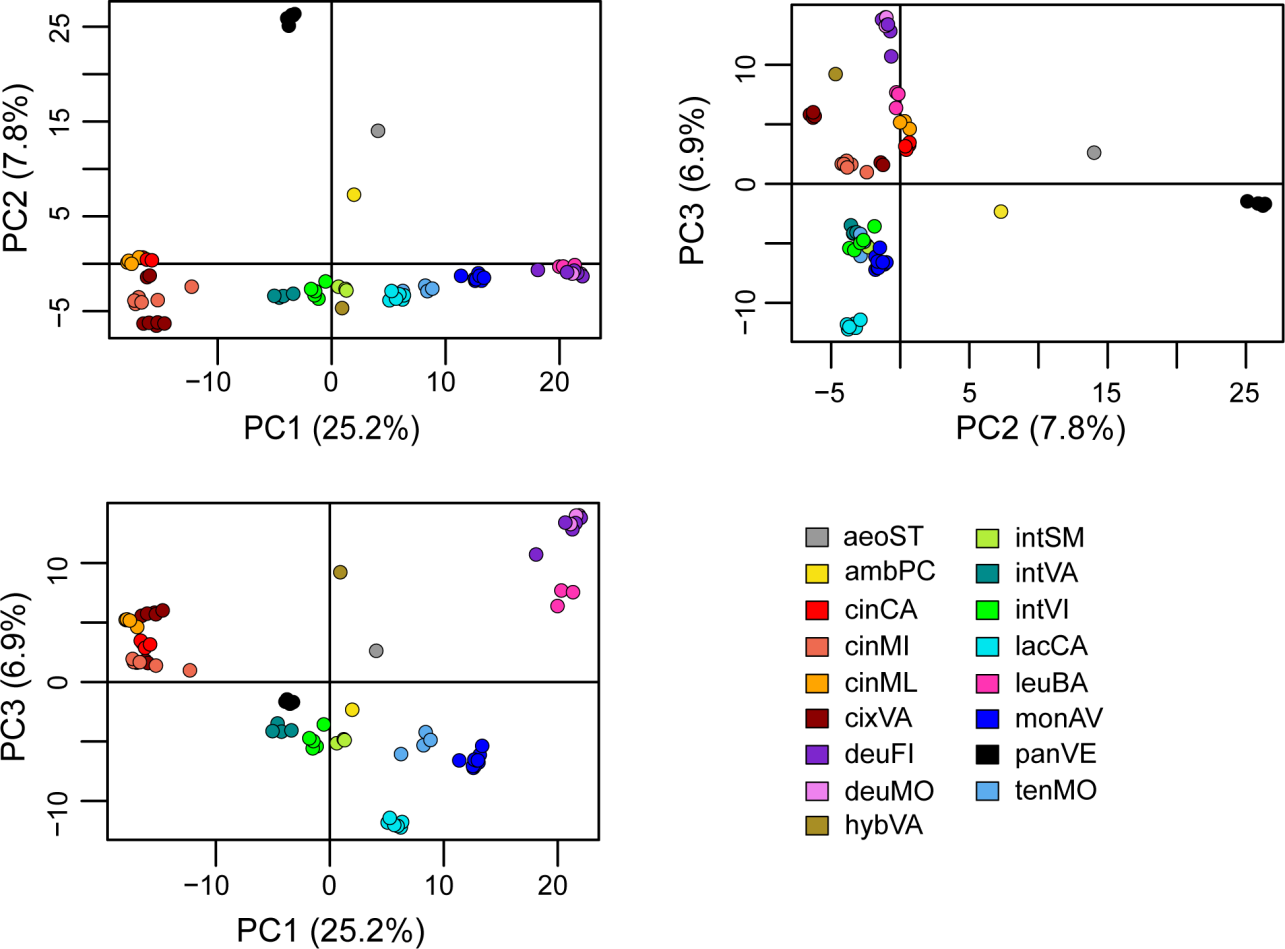
PCA analysis. For explanation of legend see **Table 1**.

The PCA explained the 39.9% of the variance along the three main axes (PC1 = 25.2; PC2 = 7.8%; PC3 = 6.9%) and showed that all the operational taxonomic units (OTUs) from the same population clustered together (**Figure 2**), with the partial exception of two samples from cixVA. *Centaurea pandataria* was distant from all the other populations, and therefore can be considered as the best outgroup for our system. Halfway between this group and the others, the OTUs relative to *C. aeolica* and *C. ambigua* subsp. *ambigua* are localized. The three microspecies of the *C. tenorei* group are very close and can be distinguished only in one projection (PC1/PC3); in all cases, they are very close to the intermediate populations (intSM, intVA, intVI), which effectively are halfway between them and the *C. cineraria* group (**Figure 2**). No difference was found among individuals of monAV. OTUs representing *C. deusta* are consistently distinct from the others.

**Figure 3:**
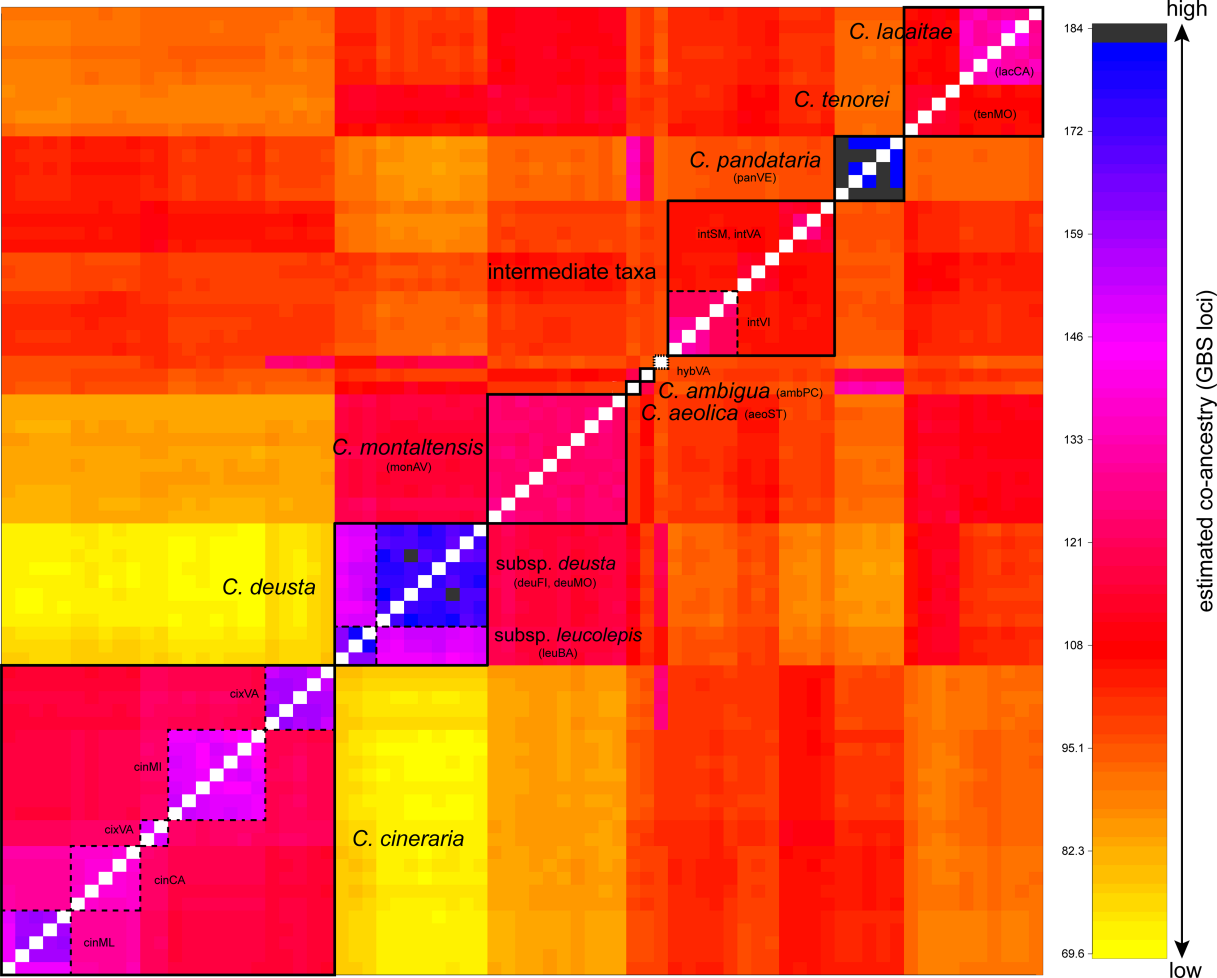
Co-ancestry plot from fineRADstructure analysis. Boxes with dashed lines highlight population samples or subspecies, while boxes with filled lines indicate well-defined groups. Co-ancestry levels increase from yellow to black.

The co-ancestry matrix resulting from the fineRADstructure analysis (**Figure 3**) showed a complex pattern of gene flow in *C. tenorei* s.l. For instance, *C. montaltensis* had high co-ancestry with the neighboring *C. deusta* (subsp. *deusta* and subsp. *leucolepis*), as well as with *C. tenorei* and *C. lacaitae* and, to a lower extent, with the intermediate populations. These latter ones had comparable levels of ancestry with *C. tenorei* s.l. and *C. cineraria*; *C. cineraria* showed high co-ancestry with *C. pandataria* and some individuals from intVI and intSM. Within *C. cineraria*, the sampled populations resulted highly structured: we observed four distinct sub-clusters, corresponding to cinCA, cinML (these two very close each to the other), cinMI, and cixVA (**Figure 3**). The two subspecies of *C. deusta* have, as expected, high co-ancestry, as well as *C. aeolica* and *C. pandataria*. The relationships of *C. ambigua* are not clear: the highest co-ancestry is here found with *C. tenorei* s.l. and *C. pandataria*.

**Figure 4:**
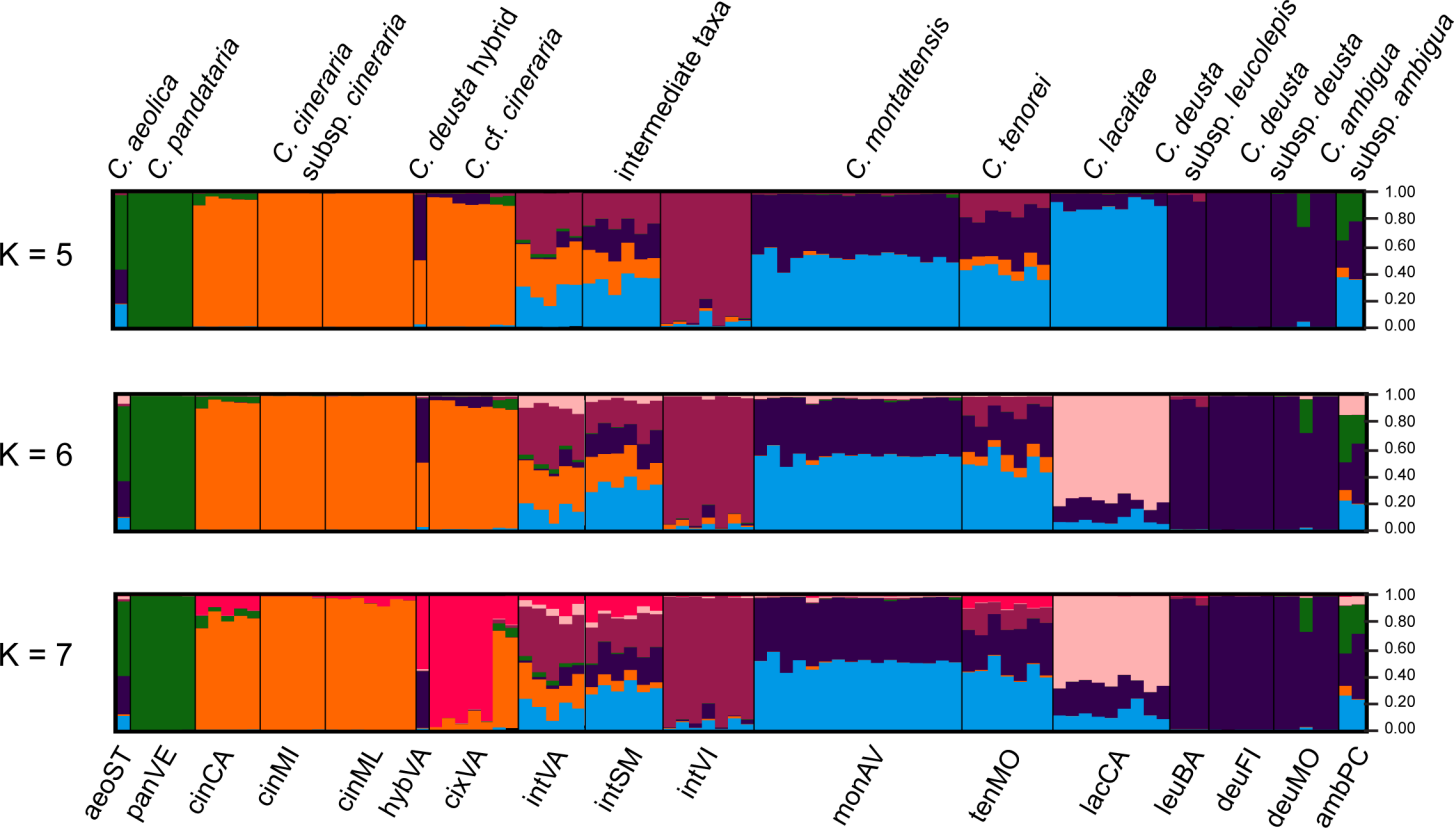
STRUCTURE plots. The analysis was carried out using 96 individuals and a selected panel of 267 SNPs.

STRUCTURE analysis indicated K = 7 as the optimal number of clusters (**Supplementary Figure 4**). Due to their possible biological relevance, other genetic clusters, i.e., K =4, K = 5, and K = 6, were also displayed in **Figure 4**. Indeed, K = 4 provided the simplest biological explanation for the genetic structure: a first group including *C. aeolica* and *C. pandataria* (aeoST and panVE), a second group encompassing *C. cineraria* (cinCA, cinMI, and cinML) and specimens resembling it (cixVA), a third group with *C. deusta* (deuFI, deuMO, and leuBA), and a last one including populations attributable to *C. tenorei* s.l. (lacCA, monAV, and tenMO) and its intermediates (intSM, intVI, and intVA). The individual labelled as hybVA was confirmed as a hybrid between *C. cineraria* and *C. deusta*. At K = 5, the coastal population intVI was further separated from the others; similarly, at K = 6, the other coastal population, lacCA was segregated from the *C. tenorei* aggregate. At K = 7, the population cixVA was separated from the other *C. cineraria* samples. As a consequence, the specimen hybVA, which is sympatric with population cixVA, is obviously a hybrid between the latter population and *C. deusta*. For all Ks, *C. montaltensis* and *C. tenorei* appeared very similar to each other and remarkably more admixed with *C. deusta* than all the other populations of the complex.

F_ST_ analysis (**Supplementary Table 5**) showed that between *C. montaltensis* and *C. tenorei* (0.092) a low genetic differentiation is present, altogether comparable to that between the two populations of *C. deusta* subsp. *deusta* (0.089). A slightly higher differentiation is observed between lacCA and monAV/tenMO (0.178 and 0.191, respectively). The plants with intermediate features (intVA, intSM, intVI) had F_ST_ values < 0.25 with *C. tenorei* s.l., with the only exception of lacCA vs. intVI (0.277); genetic structure was higher when comparing it with that of *C. cineraria* (~0.35) and *C. deusta* (~0.48). Within *C. cineraria*, the lowest differentiation was found between the populations from cinCA and cinML (geographically near), a result also evident in the fineRADstructure analysis.

## Discussion

### Ploidy, speciation, and diversification in *Centaurea tenorei* group

Despite current taxonomy distinguishes three microspecies within *Centaurea tenorei* s.l. also on account of their different ploidy (Peruzzi and Scoppola, 2008; Santangelo et al., 2017), our results show that all the taxa of this group are consistently tetraploid. In fact, after five direct counts (**Table 2**) and 50 NGS estimations in this group (**Supplementary Table 1**) we detected or postulated only 17 triploids in the group, and no diploids. Tetraploids were first indicated by Peruzzi and Cesca (2002) for *C. montaltensis* and *C. lacaitae*. However, the same authors reported as diploids a population “near Scala and Ravello” and another from “Mt. Sant’Angelo a Tre Pizzi” (*C. tenorei*). It is to be noted that the latter population corresponds to the tetraploid tenMO, whereas we could not find the former after several field trips. On Mt. Molare, we found diploid only the individuals belonging to *C. deusta* subsp. *deusta*, which is sympatric in that locality with *C. tenorei* and is widespread also elsewhere. Therefore, contrary to what previously supposed (Peruzzi and Scoppola, 2008; Pignatti, 2018), the remarkable morphological variability of the group is independent of ploidy. Such variability is especially surprising in a narrow endemic, and it is not shared with any other Campanian endemic (Del Guacchio et al., 2020b). If on one hand, previous scholars (Lacaita, 1922; Guadagno, card-index; Barone Lumaga and Menale, 2000) indicate that this variation has a large environmental component (especially regarding the vegetative features), on the other hand, STRUCTURE analysis (**Figure 4**) reveals that this variability is mainly due to the genetic influence of *C. cineraria* and *C. deusta*. Hybridization and introgression are obvious responsible of great variability in plants in general and also in *Centaurea* (López-Pujol et al., 2012; Mameli et al., 2014; Requena et al., 2020). Hybridization phenomena in *C. tenorei* s.l. were already hypothesized by several authors founding on morphological observations (Lacaita, 1922; Guadagno, 1932; Del Guacchio et al., 2022), but a definitive proof was still lacking. In this regard, they pointed especially to *C. cineraria* and considered hybrids between *C. tenorei* s.l. and *C. deusta* as rare. In particular, Guadagno (1932) considered *C. tenorei* s.l. the stabilized hybrid between *C. dissecta* Ten. (= *C. ambigua* s.l.) and *C. cineraria*. Unexpectedly, our results revealed a greater involvement of *C. deusta*: according to STRUCTURE and fineRADstructure (**Figures 3** and **4**), admixture of *C. deusta* occurs throughout the populations ascribed to our study group and its intermediates, with the partial exception of intVI, more influenced by *C. cineraria* (**Figure 4**, K =4). Despite the clinal distribution of monAV from 600 to 1000 m a.s.l., this population was genetically homogeneous. Half of the allelic pool of monAV is shared with *C. deusta* subsp. *deusta*. Marginally, we note that this parentage was possibly perceived by Lacaita (1922), who described the basal leaves of *C. tenorei* as “somewhat resembling that of *C. deusta*” and essentially invariant throughout the different populations examined by him. Moreover, he regretted that this useful feature was difficult to compare because the basal leaves wither at flowering time in this and in allied species. Even if the two subspecies of *C. deusta* do not differ in their allelic pattern, we exclude that the tetraploid *C. deusta* subsp. *leucolepis* is involved in the current gene flow with *C. tenorei* s.l., because of geographic distance. On the contrary, the only literature tetraploid count known for *C. cineraria* subsp. *cineraria* (Damboldt and Matthäs, 1975) is from a locality (“Rocky slope at a lookout point coastal road about 5.7 km west of Positano”), where intermediate populations, which resulted tetraploid in our analysis, may occur.

These considerations entail an intriguing issue: as *C. tenorei* s.l. is tetraploid, why we observe such a wide gene flow involving both *C. cineraria* (2×) and *C. deusta* subsp. *deusta* (2×)? This is difficult to explain by frequent hybridizations between these two latter taxa and diploid individuals of *C. tenorei* s.l., (Peruzzi and Cesca, 2002) because these latter must be very rare (and indeed were not found by us). Equally unlikely is the hypothesis of a tetraploid ancestor of the group, later hybridized with diploid taxa, as this would not justify the current tetraploid populations. The only parsimonious hypothesis is that a diploid ancestor of *C. tenorei* s.l. was sufficiently compatible with both *C. cineraria* and *C. deusta* to produce homoploid hybrids. The obvious consequence of this scenario would be progressive introgression of hybrid populations towards the more abundant diploid parentals, and the final dilution of the gene pool of the postulated ancestor. However, if some of these hybrids developed into autotetraploids, may have originated established hybridogenous populations. Polyploidy, in fact, is unanimously regarded as one of the driving forces of plant evolution and polyploids (autotetraploids, in our case) perform better in colonizing new habitats (Soltis et al., 2016; Becher et al., 2020). This can easily explain both the morphological variability of *C. tenorei* s.l., and its wide altitudinal range (from 1400 m to 10 m a.s.l.), a plasticity shared with *C. deusta*.

The origin of the postulated ancestor of *C. tenorei* s.l. remains obscure and might be clarified only by an extensive study involving the allied taxa of the group; this in turn is a rather insidious issue, because the obscure phylogeny of the section does not allow to identify them easily (Hilpold et al., 2014b). However, in our opinion, the hypothesis first suggested by Guadagno (1932) about the involvement of *C. ambigua* s.l. or of a taxon allied to it, remains the most suggestive. In fact, *C. ambigua* s.l. and *C. tenorei* s.l. share unquestionable morphological similarity (Pignatti and Lausi, 1982). Moreover, *C. ambigua* s.l. is geographically nearest to *C. tenorei* s.l. among the similar species (Lacaita, 1922); in this respect, we mention that it has been recently collected by us on Lago Matese (northern Campania), and possibly it occurs even southward (Terracciano, 1874; PFI [Portale della Flora d’Italia], 2022, sub *C. dissecta*). It has to be noted, however, that the most widespread subspecies, i.e., *C. ambigua* subsp. *ambigua*, as far as it is known, is tetraploid (**Table 2**).

The allelic frequencies are substantially identical in each population of *C. tenorei* s.l. (intermediates included) but different across them (**Figures 3** and **4**). This fact suggests that most of the gene flow occurred in the past and present-day populations have been founded by parentals with different introgression degrees. This is clearly reflected by the scattered distribution of the populations of the group and by their morphological distinctness, which induced several taxonomists to split the group into several taxa, ranking from species to forms (Guadagno, card-index; Santangelo et al., 2017). A remarkable exception to this general genetic distinctiveness of single populations can be found in the locality called “Valico di Chiunzi”, a pass in the hills between the northern and the southern slopes of the Peninsula of Sorrento, and therefore a natural way of passage. Incidentally, we note that it is densely traveled by man, who doubtlessly facilitated the contact of different populations by preparing new paths in the slopes, digging caves and building towers (which are preferred habitats of these plants). It is suggestive that in this locality, we find two different genetic pools of *C. cineraria*: the most common is private to that population, whereas two individuals share more affinity with the other populations of the same taxon (**Figure 4**). At Valico di Chiunzi, hybridization has been documented since a century ago (Guadagno, 1932; Del Guacchio et al., 2020a) and is still very active, involving *C. deusta* subsp. *deusta*, *C. tenorei* s.l., and *C. cineraria* subsp. *cineraria* (**Figures 3** and 4). We can add that, according to literature (e.g., Pignatti and Lausi, 1982), the natural range of *C. cineraria* is strictly linked to the coast. More precisely, Guadagno (card-index) did not observe it above 350 m a.s.l., even if he gathered on the pass as well. The plant might have been overlooked by him or confused with *C. tenorei* s.l. but this seems unlikely. Indeed, we cannot exclude that *C. cineraria* has reached the pass only recently, possibly also driven by climate change. The altitudinal shift upward has been documented for several mountain plants (Lenoir et al., 2008; Telwala et al., 2013; Zu et al., 2021) but, to the best of our knowledge, not well known in coastal plants.

### Taxa delimitation in *C. tenorei* s.l.

The populations ascribed to *C. tenorei* s.l. (i.e., tenMO, monAV, lacCA) and those indicated as ‘intermediate’ (i.e., intVI, intSM, intVA) can be grouped in the PCA graphs (**Figure 2**, especially in the PC1\PC3), but with a rather dispersed pattern. In detail, tenMO and monAV, i.e., the typical populations of *C. tenorei* and *C. montaltensis* respectively, are very close (**Figure 2**). The F_ST_ value between tenMo and monAV (0.092, **Supplementary Table 5**) is lower than the average value found between other plant species and in the average for different varieties (Huang et al., 2020; Chambers et al., 2021); also for the other taxa of *C. tenorei* s.l. and its intermediate forms, F_ST_ values are in agreement with subspecific ranks in other studies (e.g., Yu et al., 2022). The genetic structure of the two populations, however, is not identical, because that of *C. tenorei* is more complex (**Figure 4**). Morphologically, *Centaurea tenorei* and *C. montaltensis* are very weakly distinguishable (Lacaita, 1922; Peruzzi and Scoppola, 2008; Santangelo et al., 2017, **Table 1**), and several scholars do not recognize any taxonomic distinctness (e.g., Fiori, 1927). Moreover, the presumed difference about the ratio between the pappus and the cypsela, as well as the color of the bracteal ciliae vary independently within several population (Guadagno, card-index; pers. obs.), while the head width of *C. montaltensis* is fully included in the range of *C. tenorei*. Therefore, after our result on the tetraploid chromosome number shared by both taxa, distinctness of *C. montaltensis* does not seem furtherly sustainable. The population lacCA appears as more distinct, what would suggest in turn a taxonomic recognition. In this regard, we note that, according to our results, lacCA is one of the least admixed populations, and maybe the one preserving the plesiomorphic features of the unknown ancestor. However, traces of the genetic contribution by *C. deusta* are present as well (**Figure 4**). More importantly, several other similar populations included in *C. lacaitae* occur along the coasts (Santangelo et al., 2017), and we suspect that their inclusion in our study would have furtherly diluted the genetic isolation of lacCA. For these reasons, any taxonomic distinction of the typical *C. lacaitae* from *C. tenorei* results problematic and should be considered with caution. It is to be noted that *C. lacaitae*, corresponding to *C. dissecta* Ten. var. *maritima*, as circumscribed by Lacaita (1922) and recent authors (Peruzzi and Scoppola, 2008; Santangelo et al., 2017) includes not only the typical population lacCA, but also the ‘intermediate’ ones (intVI, intSM, intVA), formerly distinct as *C. dissecta* var. *maritima* f. *cinerarioides* Lacaita ex Fiori. Guadagno (card-index), for example, did not accept var. *maritima* as a separate taxon, but was aware that f. *cinerarioides* would deserve recognition.

### Centaurea deusta

According to all results, the populations ascribed to *C. deusta* group together, also including the subsp. *leucolepis*. This latter remarkable subspecies differs from the typical *C. deusta* on account of a combination of morphological features independently and sporadically occurring also in the subsp. *deusta* (i.e., pale, muticous involucral bracts, whitish flowers, often fleshy leaves), of the maritime ecology and tetraploid chromosome number (Del Guacchio et al., 2022). Hilpold et al. (2015, sub *C. corensis*) suggested an allopolyploid origin of this taxon. Nevertheless, our results better support a direct differentiation from *C. deusta* by local autotetraploidy as an adaptation to coastal environments (see also Del Guacchio et al., 2022). In fact, even at highest K values in the Structure analysis (**Supplementary Figure 5**), the genetic structure of the investigated population (leuBA), is very homogeneous and apparently not different from that of two separate mountain populations of *C. deusta* subsp. *deusta* (deuMO, deuAV). Taxonomically, the results of the co-ancestry analysis with fineRADstructure (**Figure 3**) and of PCA (**Figure 2**) concur with the subspecific rank already proposed by Del Guacchio et al. (2022) on morphological grounds.

### New hybrids

Hybrids between the homoploid taxa *C. cineraria* s.l. and *C. deusta* subsp. *deusta* seem very rare, despite the natural range of *C. cineraria* (south-western coasts of Italy) is entirely included in that of the other species (Italy, Switzerland, and Balkan peninsula). Indeed, a single hybrid between them is documented here for the first time (the individual labelled as hybVA). We may presume that *C. deusta* and *C. cineraria*, belonging to two different subsections (Hilpold et al., 2014a), are poorly compatible and not closely related. This, however, might be the consequence of the slightly different habitat of the two species, or of overlooked hybrids. The results concur with the hypothesis by Del Guacchio et al. (2022) about the hybrid between *C. tenorei* (mainly tetraploid) and *C. deusta* subsp. *deusta*, i.e., *C.* ×*cavarae* Guadagno ex Del Guacchio, Cennamo & Caputo, only known for a gathering in the same place (Valico di Chiunzi). A form morphologically similar to *C.* ×*cavarae* still occurs in its *locus classicus*. Further and finer analyses are required to assess the precise identity of these forms.

## Conclusion

This study demonstrates that the taxa belonging to the *C. tenorei* aggregate, i.e., *C. tenorei*, *C. lacaitae*, and *C. montaltensis*, including the intermediate forms, are chiefly tetraploid and that the current recognition of three microspecies is not supported by genetic data. These taxa appear of hybridogenous origin, with an important contribution of *C. deusta* (or its ancestor) and, to a lesser extent, *C. cineraria*. Some populations of this aggregate, however, have unique genetic features and may be regarded as evolutionary significant units (ESUs). We also find that the locality “Valico di Chiunzi” is particularly interesting from the evolutionary and ecological point of views because it harbors populations of *C. cineraria* out of their ecological range that hybridize with *C. deusta* and *C. cineraria*, creating an intricate pattern of relationships across species. Furthermore, this study confirms that SNPs markers are a powerful tool to analyze the genetic structure of difficult plant systems where mixed-ploidy, hybridization and introgression occur and to determine ploidy at individual level in the absence of chromosome counts.

## Data availability statement

The raw Illumina sequences used in the study are available in the Sequence Read Archive (SRA) repository under NCBI BioProject PRJNA928762. Voucher specimens are deposited at the Herbarium of the Botanical Garden of Naples (NAP) and available upon request. Supporting information to the paper is available as **Supplementary Material**.

## Author contributions

EDG and PCE contributed to conception and design of the study. Mainly EDG and PCE field-collected plant material, but all authors collected at least in one locality. DL carried out molecular work and data analysis. LP was responsible for chromosome counts. DDL, EDG, and PCA wrote the first draft of the manuscript. All authors contributed to the article and approved the submitted version. PCA supervised the whole work.
