# Supplementary figures and images for: Genotyping-by-sequencing provides new genetic and taxonomic insights in the critical group of Centaurea tenorei
Source: Front Plant Sci. 2023 May 16;14:1130889. doi: 10.3389/fpls.2023.1130889 (PMC10228698; doi:10.3389/fpls.2023.1130889)

Supplementary Figure 1. Observed heterozygosity per locus.

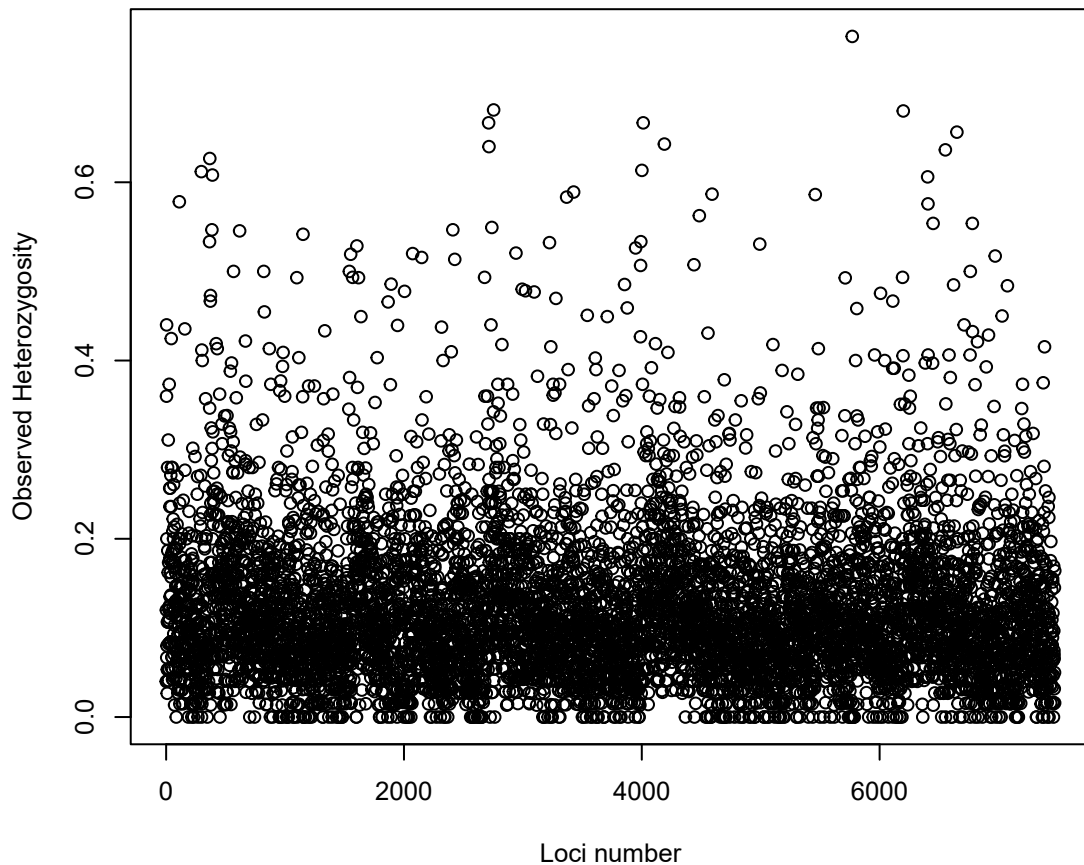

Supplement: Supplementary file 1 [file DataSheet_1.pdf]

Supplementary Figure 3. Distribution of allele frequencies.

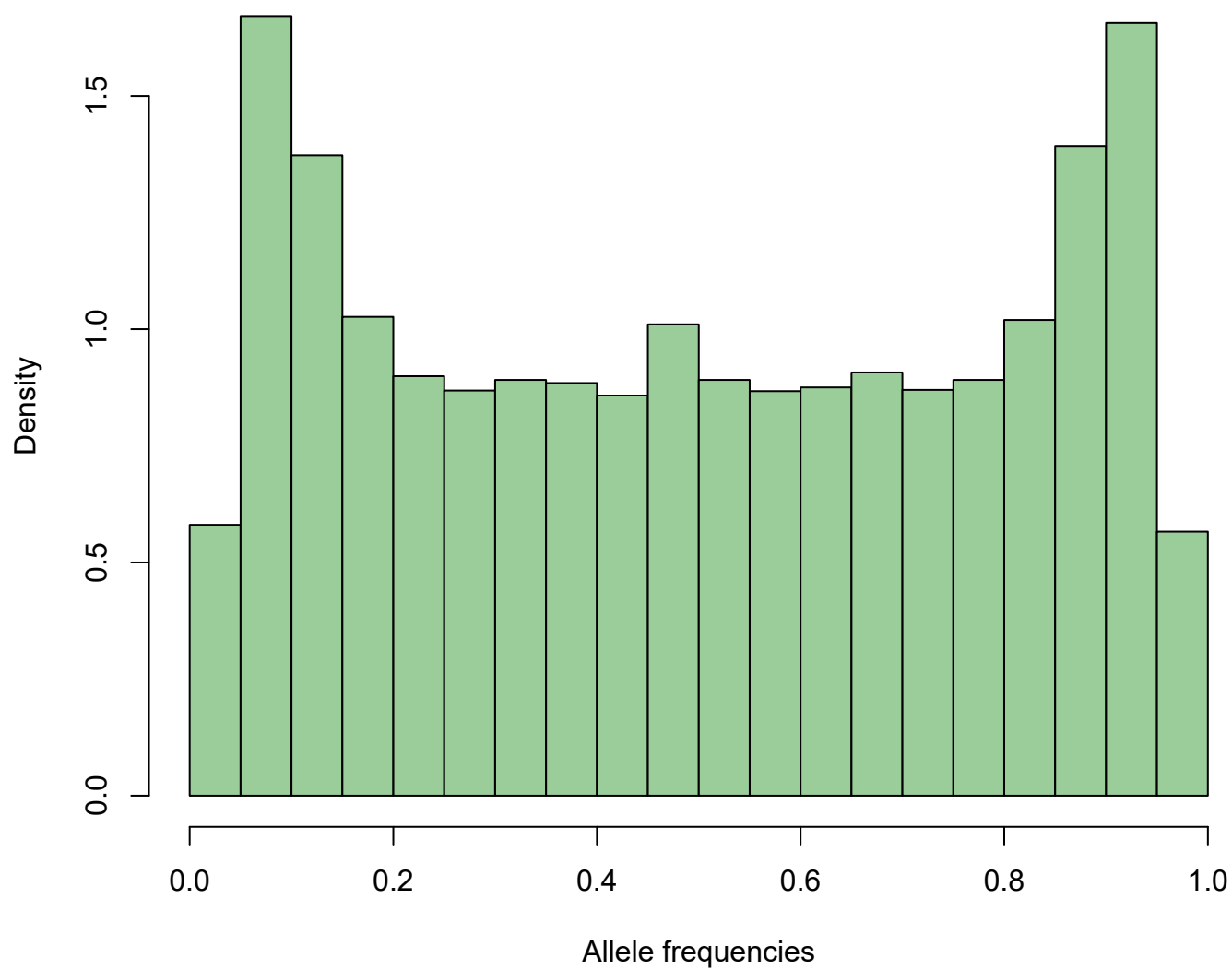

Supplement: Supplementary file 3 [file DataSheet_3.pdf]

Supplementary Figure 5. Results of STRUCTURE analysis from K = 2 to K = 12.

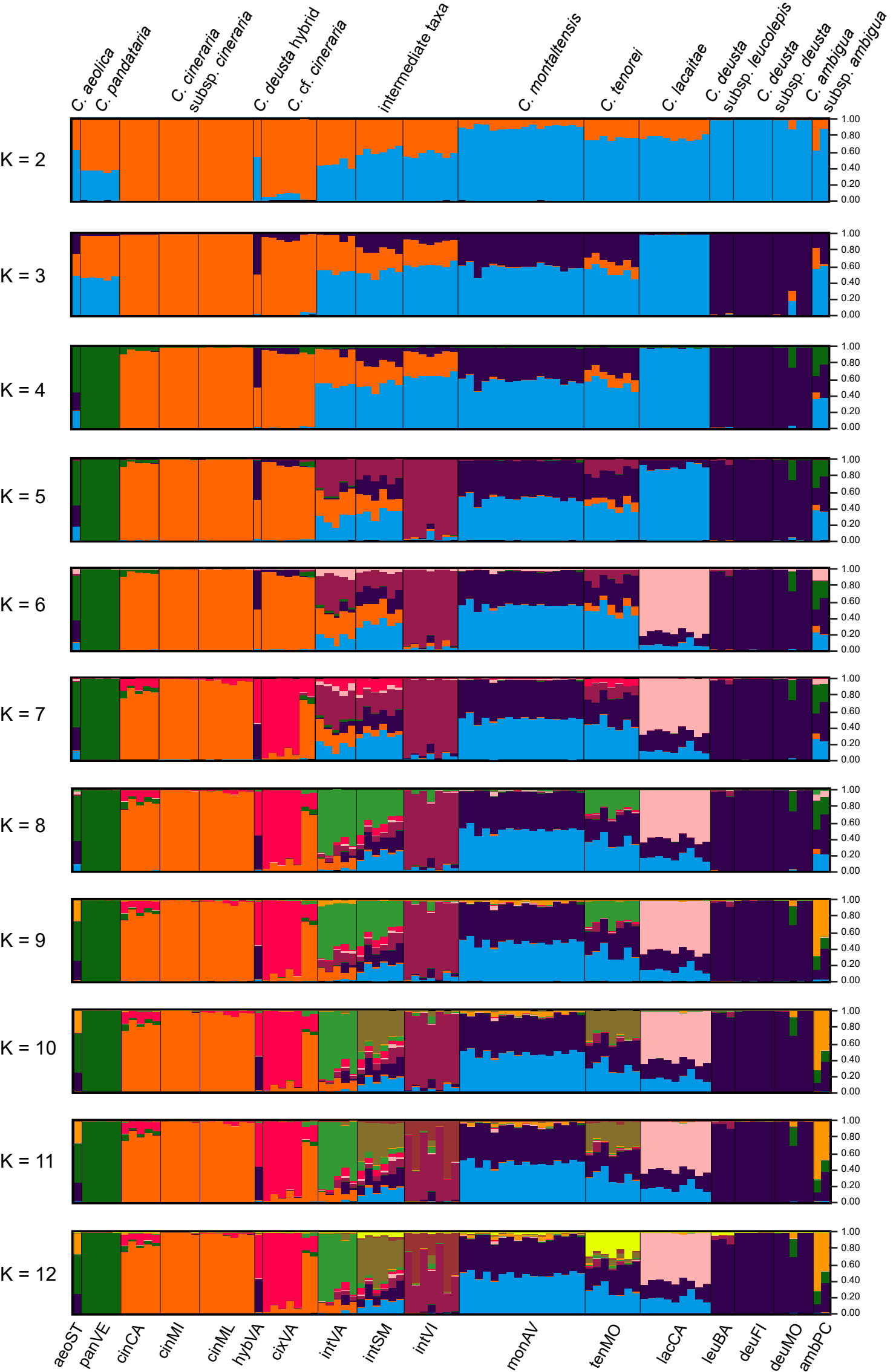

Supplement: Supplementary file 5 [file DataSheet_5.pdf]
