# Supplementary material for: Genotyping-by-sequencing provides new genetic and taxonomic insights in the critical group of Centaurea tenorei
Source: Front Plant Sci. 2023 May 16;14:1130889. doi: 10.3389/fpls.2023.1130889 (PMC10228698; doi:10.3389/fpls.2023.1130889)

Supplementary Figure 2. Expected heterozygosity as function of observed heterozygosity per locus.

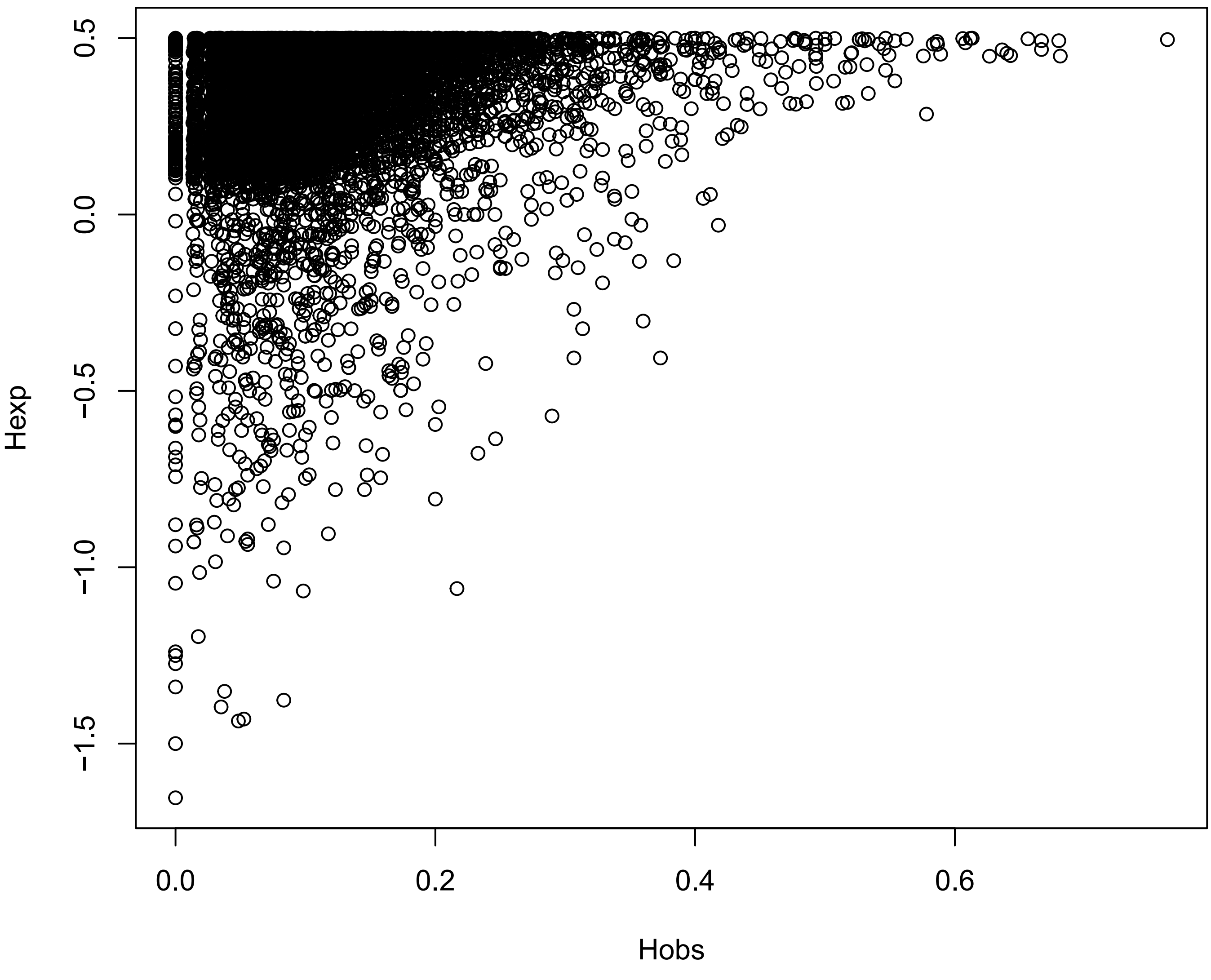

Supplement: Supplementary file 2 [file DataSheet_2.pdf]
