# Supplementary material for: Genotyping-by-sequencing provides new genetic and taxonomic insights in the critical group of Centaurea tenorei
Source: Front Plant Sci. 2023 May 16;14:1130889. doi: 10.3389/fpls.2023.1130889 (PMC10228698; doi:10.3389/fpls.2023.1130889)
Supplement: Supplementary file 4 [file DataSheet_4.pdf]

Supplementary Figure 4. Plot of mean Ln probability of data for each K of STRUCTURE analysis and Evanno table.

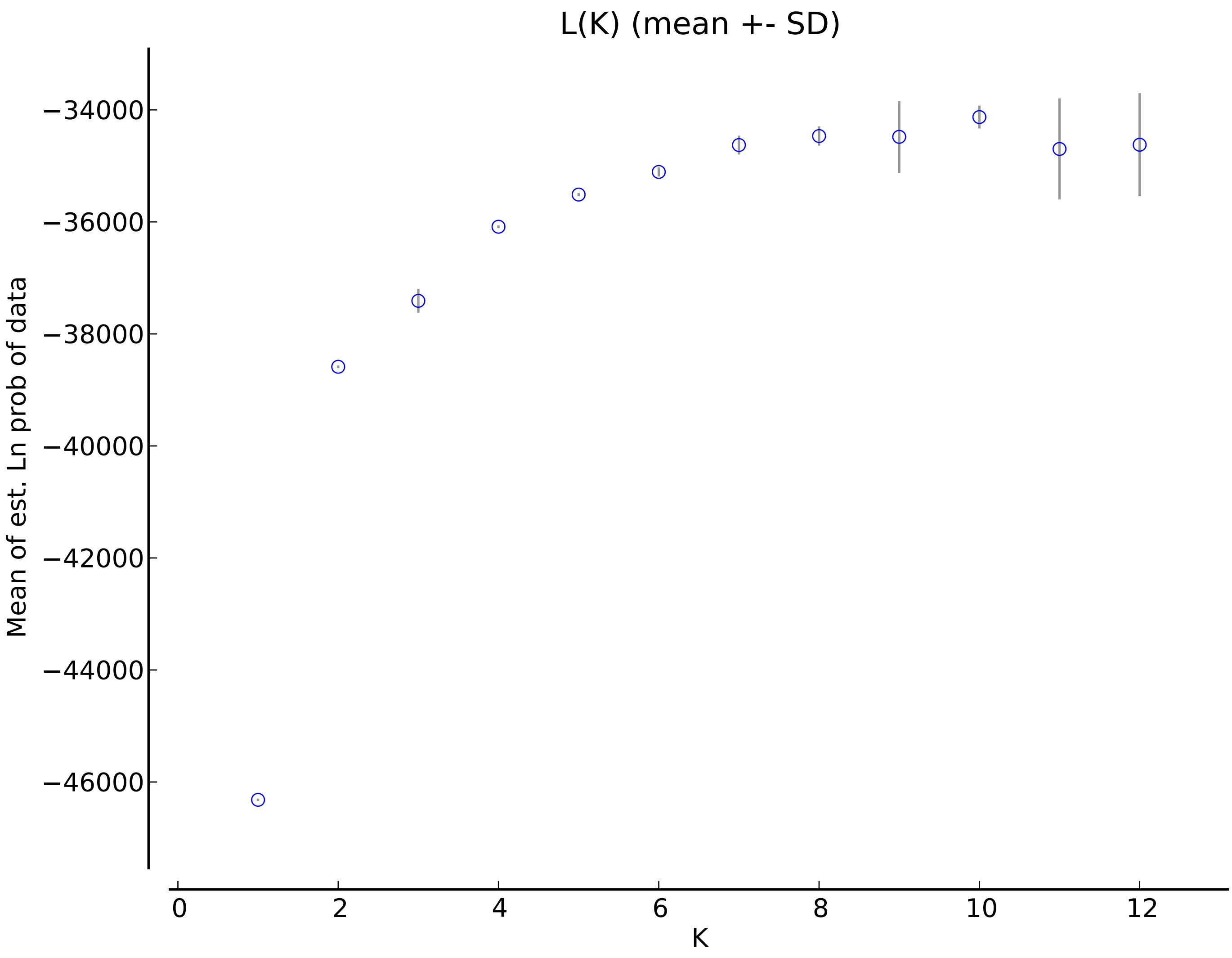

K	Reps	Mean LnP(K)	Stdev LnP(K)	Ln'(K)	|Ln''(K)|	Delta K
1	10	-46318.390000	0.606355	—	—	—
2	10	-38586.410000	1.250289	7731.980000	6554.720000	5242.564525
3	10	-37409.150000	190.250643	1177.260000	146.030000	0.767566
4	10	-36085.860000	3.625895	1323.290000	749.320000	206.657969
5	10	-35511.890000	6.443507	573.970000	170.120000	26.401773
6	10	-35108.040000	59.854921	403.850000	76.870000	1.284272
7	10	-34627.320000	147.399222	480.720000	320.010000	2.171043
8	10	-34466.610000	149.987255	160.710000	175.140000	1.167699
9	10	-34481.040000	621.279361	-14.430000	367.590000	0.591666
10	10	-34127.880000	182.318237	353.160000	922.970000	5.062412
11	10	-34697.690000	880.433025	-569.810000	645.340000	0.732980
12	10	-34622.160000	898.313989	75.530000	—	—
